# Emerging roles of long non-coding RNAs in tumor metabolism

**DOI:** 10.1186/s13045-018-0648-7

**Published:** 2018-08-22

**Authors:** Hui Sun, Zhaohui Huang, Weiqi Sheng, Mi-die Xu

**Affiliations:** 1 Department of Pathology, Fudan University Shanghai Cancer Center, Shanghai, 200032 China; 2 Wuxi Cancer Institute, Affiliated Hospital of Jiangnan University, Wuxi, Jiangsu China; 3 Department of Pathology, Tissue bank, Fudan University Shanghai Cancer Center, Shanghai, 200032 China

**Keywords:** lncRNAs, Tumor, Metabolism, Reprogramming, Dysregulation

## Abstract

Compared with normal cells, tumor cells display distinct metabolic characteristics. Long non-coding RNAs (lncRNAs), a large class of regulatory RNA molecules with limited or no protein-coding capacity, play key roles in tumorigenesis and progression. Recent advances have revealed that lncRNAs play a vital role in cell metabolism by regulating the reprogramming of the metabolic pathways in cancer cells. LncRNAs could regulate various metabolic enzymes that integrate cell malignant transformation and metabolic reprogramming. In addition to the known functions of lncRNAs in regulating glycolysis and glucose homeostasis, recent studies also implicate lncRNAs in amino acid and lipid metabolism. These observations reveal the high complexity of the malignant metabolism. Elucidating the metabolic-related functions of lncRNAs will provide a better understanding of the regulatory mechanisms of metabolism and thus may provide insights for the clinical development of cancer diagnostics, prognostics and therapeutics.

## Background

Metabolism is the set of life-sustaining chemical transformations within the cells of organisms. Most of the structures that make up living organisms are made from three basic classes of molecule: carbohydrates, lipids, and amino acids. The metabolic pathways in the human body focus either on making these molecules during the construction of cells and tissues or on breaking them down by digestion and using them as a source of energy. Malignancy is a disease characterized by the evasion of cell proliferation checkpoints. In general, tumor cells metabolize glucose, glutamine, and fatty acids (FAs) at much higher rates than their normal counterparts. The metabolic ecology of tumors is complex, and tumor cells undergo fundamental changes in metabolic pathways. Multiple molecular mechanisms converge to alter the overall cell metabolism and to provide support for the three basic needs of proliferating cells: rapid ATP generation to maintain energy status; increased biosynthesis of macromolecules; and strict maintenance of appropriate redox status [1]. To satisfy these needs, cancer cells alter the metabolism of the four major macromolecules, carbohydrates, proteins, lipids, and nucleic acids. A number of similar alterations have also been observed in normal cells that are rapidly proliferating in response to pathophysiological growth signals [2, 3].

Normal cells produce energy primarily through mitochondrial oxidative phosphorylation, which is the metabolic pathway in which cells use enzymes to oxidize organic compounds including carbohydrates, lipids, and amino acids, thereby releasing energy for the production of adenosine triphosphate (ATP) [2]. Tumor cells can satisfy the needs of their rapid and unrestrained proliferation by a high rate of glycolysis followed by lactic acid fermentation even in the presence of abundant oxygen. This process is called aerobic glycolysis, also known as the Warburg effect [4]. Aerobic glycolysis was first recognized in the 1920s by Otto Warburg, who found that cancer tissues metabolized glucose to lactate through glycolysis at an increased rate, even under normal oxygen concentrations [5, 6]. The Warburg effect has been observed in different types of tumors and has been widely accepted as a hallmark of altered metabolism in cancer cells [7]. However, these alterations are not specific to tumors, because similar changes have also been observed in rapidly proliferating normal cells [2, 3, 8].

Although glucose metabolism is important for cell proliferation, it has recently been recognized that other nutrients, such as amino acids and lipids, also significantly contribute to cell proliferation [9, 10]. “Glutamine metabolism” (i.e., accelerated glutamine intake and glutaminolysis in tumor cells) is another important energy metabolic mode of tumor cells in addition to the Warburg effect [11–14]. Like glucose, glutamine is anaplerotic, meaning that it provides energetic precursors such as oxaloacetate for the Krebs Cycle (also called the tricarboxylic acid cycle, TCA). Glycolysis and glutaminolysis increase carbon flux inside the cell, resulting in the accumulation of the Krebs cycle precursor intermediate to activate another metabolic pathway—the pentose phosphate pathway [15], which generates NADPH, reducing glutathione and enhancing the amelioration of oxidative stress. The pentose phosphate pathway also produces ribose-5-phosphate, which is essential for the biosynthesis of nucleic acids. While normal cells obtain most FAs from circulating lipids, tumor cells show increased dependence on endogenous FA synthesis to satisfy their rapacious metabolic needs [16]. Under hypoxic conditions, when glucose metabolism in the tumor is blocked, the glutamine metabolism can supply the necessary energy for tumor cell survival. In addition, tumor cells could take more acetic acid from the outside environment to supplement the acetyl-CoA supply in cells and further feed the FA synthesis pathway to promote tumor growth.

The implications of the Warburg effect have been further extended in recent years as cumulative evidence confirmed that the mode of metabolism during tumorigenesis undergoes dramatic changes involving glycolysis, the Krebs cycle, oxidative phosphorylation, amino acid metabolism, FA metabolism, and nucleic acid metabolism, among many other aspects. This phenomenon is called metabolic reprogramming. Malignant transformation involves excessive glucose uptake, lactate excretion, aerobic glycolysis, glutamine, and lipid metabolism, all of which allow cancer cells to survive in adverse microenvironments [17]. The metabolic profiles of tumors with the same genetic alterations show differences depending on the origin of the tissue, suggesting that the tissue microenvironment may affect the metabolic activity of cancer cells [18]. Elucidating the mechanism of cell metabolism reprogramming and its relationship with the occurrence and development of the tumor and developing methods for the intervention and correction of abnormal cell metabolism are promising new ideas for the diagnosis, prevention, and treatment of tumors.

**Fig. 1:**
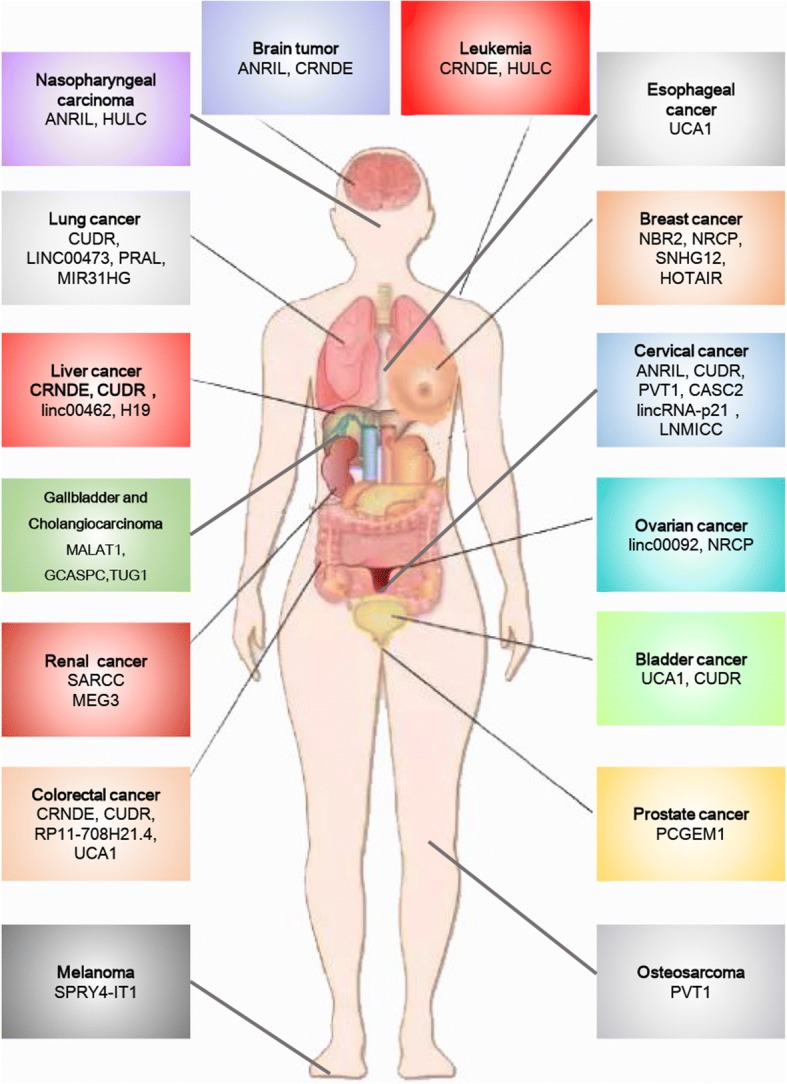
Metabolism involving lncRNAs that are dysregulated in human malignancies

**Fig. 2:**
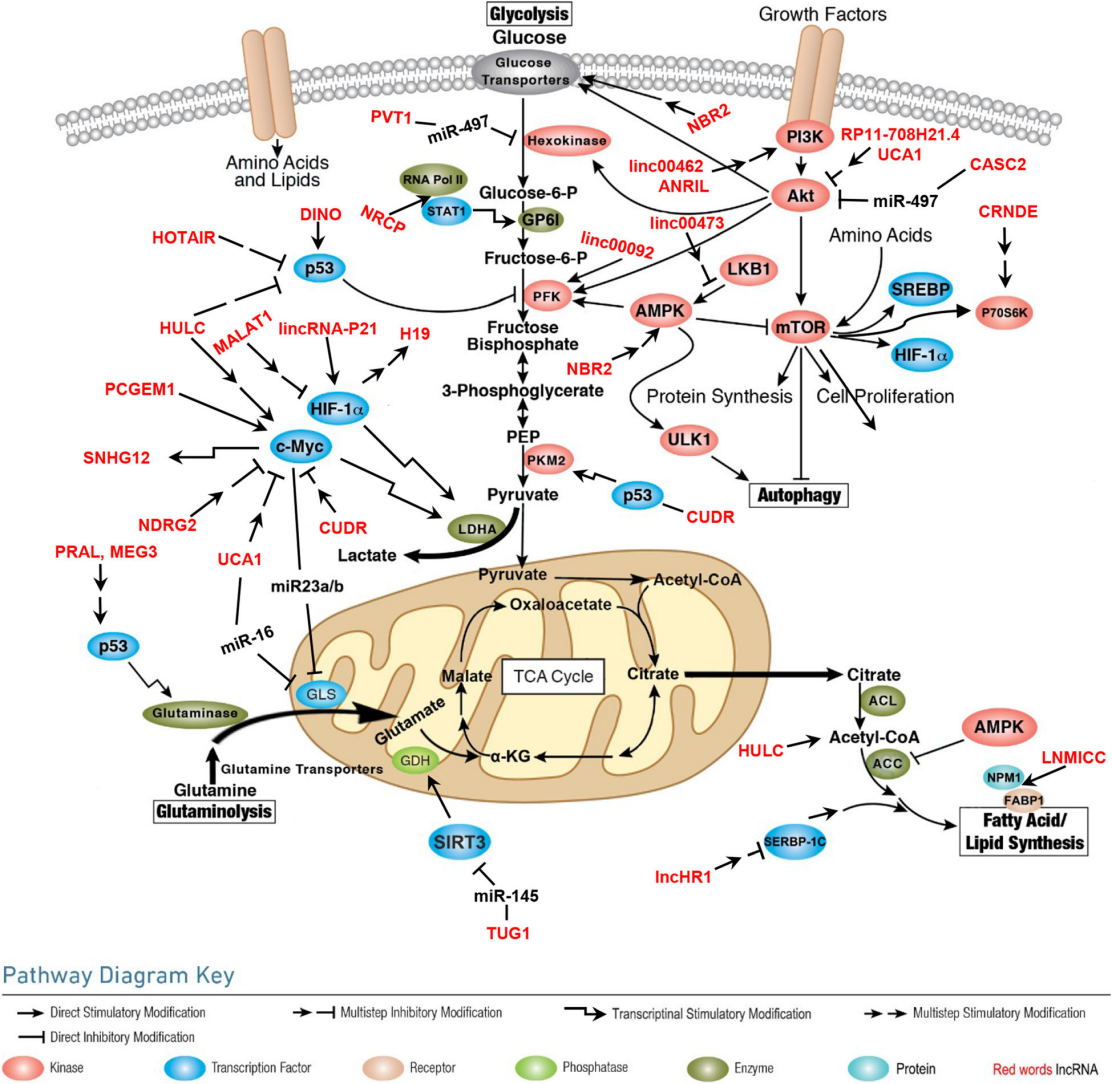
LncRNAs regulate metabolic rearrangement by targeting metabolism-related signals

**Fig. 3:**
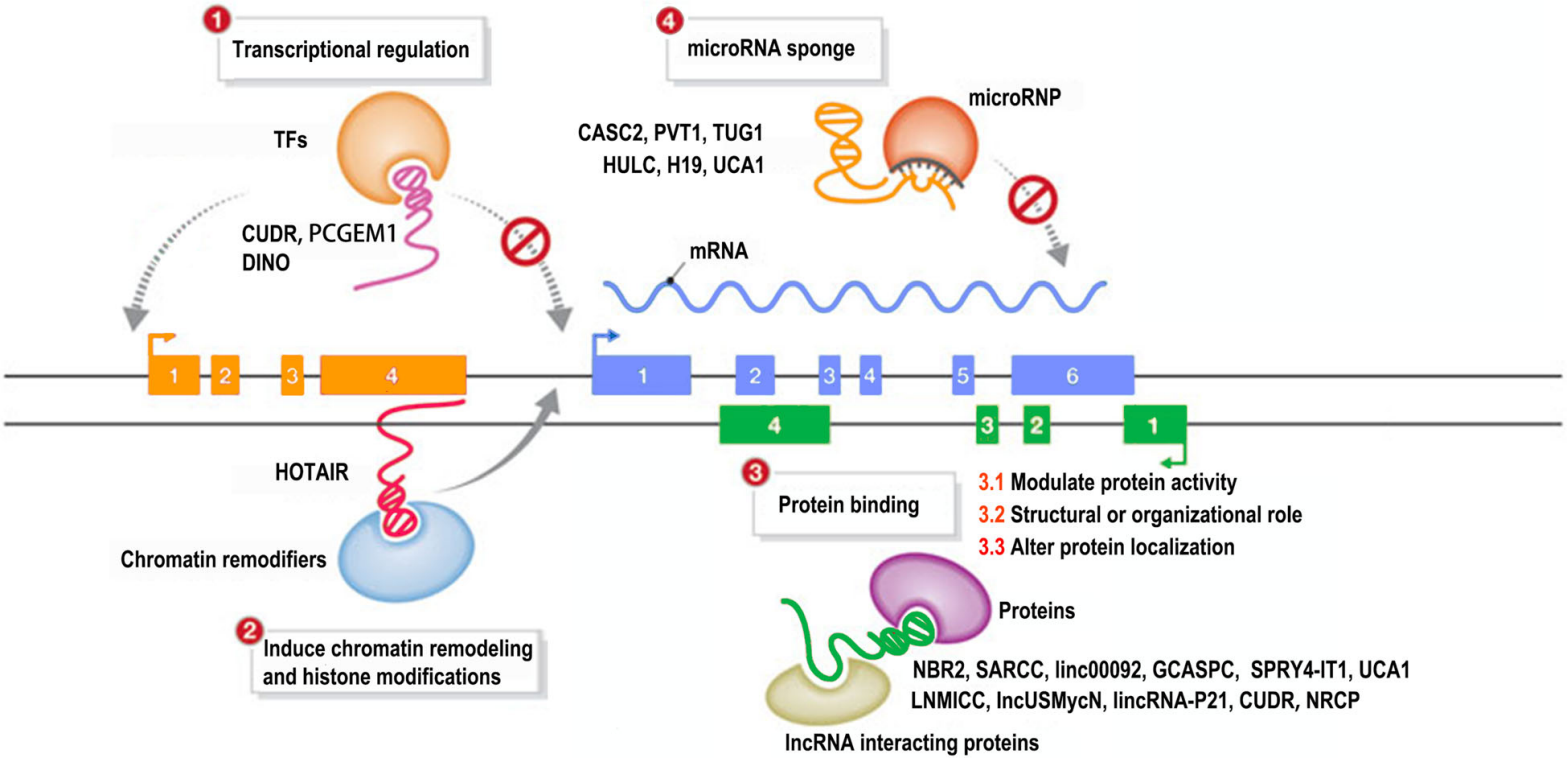
Functional mechanisms of metabolic lncRNA in tumor entities

Long non-coding RNAs (lncRNAs) are defined as mRNA-like transcripts longer than 200 nucleotides that have little or no protein-coding potential [19–21]. LncRNAs were previously regarded as “junk products” of transcription and were neglected, but recently, many lncRNAs have been identified as regulators of tumorigenesis and progression via a series of cellular processes, including cell proliferation [22, 23], apoptosis [24, 25], metastasis [26], and differentiation [27] at the transcriptional and posttranscriptional levels [28]. LncRNAs are extensively dysregulated in human malignancies and control different aspects of cellular energy metabolism, including glucose metabolism [29, 30], plasma lipid homeostasis [31], and glutamine metabolism [32] (Fig. 1), and multiple altered metabolic pathways in cancers are tightly regulated by lncRNAs [33, 34] (Figs. 2 and 3). In this review, we discuss the regulatory roles of lncRNAs in the essential metabolic rearrangement and the pathways affected in cancer.

## LncRNAs and metabolic reprogramming

### LncRNAs and glycometabolism

Malignant cells are known for accelerated nutrient and energy metabolism and increased glucose metabolism and uptake. In eukaryotic cells, glucose transport consists of two different types of membrane-related carrier proteins, namely, Na^+^-glucose linked transporters (SGLTs) and glucose transporters (GLUTs) [35]. GLUTs play a crucial role in glucose metabolism in transformed cells [36, 37]. A number of lncRNAs can regulate glycolytic steps by binding GLUTs. A novel large antisense non-coding RNA (named ANRIL) spans a 126.3 kb region and transcribes as a 3.8 kb lncRNA in the antisense orientation of the INK4B-ARF-INK4A gene cluster [16, 38, 39]. ANRIL is upregulated in nasopharyngeal carcinoma (NPC). By activating the AKT/mTOR signal, ANRIL upregulates GLUT1 and LDHA expression, thus increasing glucose uptake and promoting cancer progression in NPC cells [40]. Adenosine monophosphate-activated protein kinase (AMPK) is a critical sensor of cellular energy status in eukaryotic cells, and activated AMPK functions to promote catabolic processes (such as glycolysis, FA oxidation, and autophagy) and repress anabolic processes (such as lipid, protein, and sterol synthesis), resulting in restoration of the energy balance [41]. As anabolic processes are often required to support tumor survival and growth, the AMPK signaling pathway plays tumor-suppressive roles in many types of cancers. Currently, a glucose starvation-induced lncRNA called NBR2 (the neighbor of BRCA1 gene 2) was found to be induced in cancer cells by the LKB1–AMPK pathway under energy stress conditions [42]. Intriguingly, NBR2 can in turn interact with the kinase domain of AMPKα and promote AMPK kinase activity, thus forming a feed-forward loop to potentiate AMPK activation upon glucose starvation [34]. However, under the energy stress imposed by phenformin treatment, NBR2 promotes glucose uptake by upregulating GLUT1 expression but not by affecting phenformin-induced AMPK activation [43]. Thus, NBR2 may promote cancer cell glucose uptake by participating in alterable biological processes in response to different intracellular environments. GLUT4 is directly associated with carbohydrate metabolism. It translocates to the plasma membrane and facilitates the intracellular transport of glucose under insulin stimulation [44]. Ellis et al. found that the lncRNA CRNDE, which is upregulated in colorectal tumors, whereas little to none is expressed in the normal colorectal epithelium [45], can positively regulate GLUT4 transcription and glucose intake [30].

In addition to binding to glycolysis transporters, lncRNAs can also regulate glycolytic steps by binding to key enzymes (Fig. 2). The glycolytic enzyme 6-phosphofructo-2-kinase/fructose-2,6-bisphosphatase 2 (PFKFB2) acts as a functional homodimer that catalyzes both the synthesis and hydrolysis of fructose-2,6-bisphosphate (Fru-2,6-P2). By directly binding with PFKFB2, linc00092 promotes ovarian cancer metastasis by altering glycolysis and sustaining the local supportive function of cancer-associated fibroblasts (CAFs) one of the most abundant cell components in the tumor microenvironment, which promotes carcinogenesis and cancer progression in different cancer cell types [46]. miR-17-3p belongs to the polycistronic miR-17-92 cluster, which could associate with c-Myc, a well-studied oncogenic transcription factor showing pivotal promoting effects on cellular glycolysis [47]. miR-17-3p, which targets GCASPC (gallbladder cancer-associated suppressor of pyruvate carboxylase), can suppress pyruvate carboxylase-dependent cell proliferation in gallbladder cancer by binding and destabilizing the pyruvate carboxylase (PC) protein [48]. NRCP (lncRNA ceruloplasmin) can function as an intermediary molecule by connecting STAT1 to RNA polymerase II, which leads to upregulation of the key glycolysis enzyme glucose-6-phosphate isomerase (GP6I) and thus promotes glycolysis and cancer progression [49]. Pyruvate kinase M2 (PKM2) is essential for glucose metabolism, cell proliferation, cell migration, and tumor angiogenesis because dimeric PKM2 diverts glucose metabolism towards anabolism through aerobic glycolysis [50, 51]. The lncRNA CUDR is well known to be involved in tumorigenesis. Acting as a sponge cushion linking SET1A and pRB1, CUDR increases the expression of HULC, β-catenin, TERT, and c-Myc in human liver cancer stem cells, thus initiating stem cell malignant transformation [52–54] in a metabolic paradigm; however, CUDR can form a complex with p53 to promote hepatocarcinogenesis by transcriptionally activating PKM2 [55]. Our recent work revealed that FEZF1-AS1 enhances glycolysis through binding and increasing the stability of PKM2 in CRC cells [56]. Hexokinase catalyzes the first irreversible step of glucose metabolism, thereby producing glucose-6-phosphate through the ATP-dependent phosphorylation of glucose [57]. In bladder carcinoma, lncRNA UCA1 is overexpressed and promotes glycolysis by upregulating hexokinase 2 (HK2), which promotes aerobic glycolysis and acts as the key indicator of the Warburg effect [58, 59]. LncRNA PVT1 promotes glucose metabolism in osteosarcoma by inhibiting miR-497/HK2 signaling through a competing endogenous RNA (ceRNA) mechanism [60].

The let-7/Lin28 pathway plays a central role in mammalian glucose metabolism [61]. Lin28 promotes malignancy by blocking the biogenesis of tumor-suppressive let-7 and by derepressing the expression of oncogenic lncRNA—H19 [62]. Moreover, H19 can act as a ceRNA to sponge let-7 and upregulates Lin28 expression in breast cancer cells; in turn, this induction of Lin28 expression further restricts let-7 function [62]. In addition, by directly binding to let-7, H19 can decrease let-7 availability and suppress the insulin pathway [63, 64]. Taken together, these findings suggest that H19, let-7, and Lin28 may form a glucose metabolism-related double-negative feedback loop in breast cancer cells.

#### LncRNA and lipid metabolism

Lipids are a class of water-insoluble molecules that include triglycerides, phospholipids, sterols, and sphingolipids. The main structural components of biological membranes include phospholipids, sterols, and sphingolipids, whereas triglycerides provide energy storage. Lipids not only play a pivotal role in metabolic processes but also act as signaling molecules [65]. Similar to glucose metabolism, aberrant alterations in lipid metabolism have also been observed in cancer cells [66]. Regardless of the concentration of extracellular lipids, the main synthetic pathway of FAs is de novo synthesis. Instead of increase glucose uptake, certain tumor cells could utilize increase lipid oxidation as their main energy source. For example, malignant prostate cells are characterized by a low rate of glucose uptake but a high FA intake [67].

As master regulators of hepatic lipid homeostasis, sterol regulatory element binding proteins (SREBPs) play extensive roles in lipid metabolism. Mammals have three SREBPs (-1a, -1c, and -2), of which SREBP-1a and SREBP-1c preferentially activate the genes for FA synthesis, and SREBP-2 activates the cholesterol synthesis genes [68]. LncHR1 was found to decrease lipid metabolism by repressing the SREBP-1c promoter activity and FA synthase (FAS), resulting in decreased accumulation of oleic acid-induced hepatic cell triglyceride (TG) and lipid droplet (LD) in the Huh7 human hepatoma cell line [69]. FA-binding proteins (FABP), as indispensable carriers of FA uptake and transport, have been proven to be critical central regulators of FA metabolism [70]. FABP5 is a member of the FABP family and exhibits high-affinity binding to long-chain FAs. LncRNA LNMICC can recruit the nuclear factor NPM1 to the promoter of FABP5, thus reprogramming FA metabolism and promoting lymph node metastasis in cervical cancer [71].

Acyl-CoA synthase long-chain (ACSL) family members catalyze the initial step in cellular long-chain FA metabolism in mammals; ACSL1, one of the major isoforms of the ACSL family, can increase the uptake of FAs in hepatoma cells and can be regulated by the transcriptional factor PPARα [72–74]. HULC is the first lncRNA found to be specifically overexpressed in hepatocellular carcinoma (HCC) [75]. In hepatoma cells, HULC suppresses miR-9 targeting PPARα silencing by eliciting the methylation of CpG islands in the miR-9 promoter, and PPARα activates ACSL1 transcription. In this case, HULC stimulates the accumulation of intracellular triglycerides and cholesterol through the miR-9/PPARα/ACSL1 signaling pathway in hepatoma cells [76].

Fatty liver is an early manifestation of various liver toxicities and is not directly related to the occurrence of primary liver cancer. However, some of the etiologies of fatty liver, such as alcohol, malnutrition, drugs, and toxic damage, are pathogens of both fatty liver disease and liver cancer; therefore, fatty liver and hepatic cancer are often intertwined and share a common molecular regulatory mechanism. LncRNA has been potentially implicated in adipogenesis. Peroxisome proliferator-activated receptor-γ (PPARγ) is a master transcriptional regulator of adipogenesis. Insulin influences adipocyte differentiation through the regulation of MAPKs, including ERK1/2 (p44/p42), p38 and c-Jun amino-terminal kinase (JNK), each of which can regulate adipogenesis [77]. LncRNA-SRA (steroid receptor RNA) functions as an RNA coactivator for nonsteroid nuclear receptors and contributes to adipogenesis and insulin sensitivity via regulating PPARγ and P38/JNK phosphorylation [77, 78], and SRA knockout downregulates the size of adipocytes and improves glucose tolerance by protecting against high-fat diet-induced obesity and fatty liver [79]. We speculated that the SRA-PPARγ-P38/JNK pathway may also be implicated in HCC-related lipid metabolism rearrangement.

Lipin 2 is an enzyme that converts phosphatidate to diacylglycerol (DAG), and diacylglycerol O-acyltransferase 2 (DGAT2) is an enzyme involved in the conversion of DAG to triacylglycerol. LncRNA SPRY4-IT1 expression is low in normal human melanocytes but elevated in melanoma cells. By directly binding with lipin 2, SPRY4-IT1 downregulates the expression of DGAT2, acyl carnitine, fatty acyl chains, and triacylglycerol, thereby leading to cellular lipotoxicity and functioning as an oncogene in human melanoma cells, which provides novel insight into the mechanisms by which extranuclear processing of lncRNAs contributes to lipid metabolism [80].

#### LncRNA and amino acid metabolism

In addition to the rearrangement of glucose metabolism, tumor cells also exhibit accelerated glutamine intake and glutaminolysis. Recent work has shown that TUG1 increased glutamine metabolism and increased tumorigenic potential by functioning as an endogenous competing RNA (ceRNA), antagonizing miR-145 and indirectly upregulating sirtuin 3 (Sirt3) and glutamate dehydrogenase (GDH) expression, which provides evidence for its pivotal role in intrahepatic cholangiocarcinoma (ICC) [81]. Reactive oxygen species (ROS), as by-products of cell metabolism, can induce genetic and epigenetic alterations in human carcinogenesis [82]. Tumor cells depend for their survival not only on glycolysis but also on glutamine metabolism [11–13]. For instance, glutamine metabolism plays a key role in maintaining redox balance and ROS levels in tumor cells [83], and glutamine can be converted to glutamate, a precursor of glutathione, by glutaminase 2 (GLS2, a GLS enzyme catalyzing the conversion of glutamine to glutamate) during ROS-induced stress [84].

The c-Myc family are pivotal glutamine metabolism regulators that regulate the intracellular transposition of glutamate and promote the conversion of glutamine to glutamate [17, 85]. In colorectal cancer, by suppressing the expression of β-catenin and thereby decreasing c-Myc expression, the lncRNA N-Myc downstream-regulated gene 2 (NDRG2) could inhibit glycolysis, glutaminolysis, and thus cancer growth [86]. Previous data showed that UCA1 plays a tumor suppressor role by reducing the expression level of c-Myc in esophageal squamous cell carcinoma [87]. Moreover, in bladder cancer cells, by acting as a miR-16 sponge and upregulating the expression of miR-16 targeting GLS2, UCA1 contributes to glutamine metabolism and represses ROS formation in bladder cancer [32]. These reports reveal that UCA1 may implement its role in glutamine metabolism by multiple mechanisms.

#### Functions of lncRNA and mitochondria

In eukaryotic cells, mitochondria are critical hubs for the integration of several key metabolic processes (Fig. 2). To maintain homeostasis, the genome produces specific lncRNA nucleic acids and proteins to coordinate the intense cross-talk between the mitochondria and the nucleus. Currently, the concept of regulation driven by lncRNAs is extending from the nuclear and cytosolic compartments to the mitochondria. Based on the discoveries that (i) the mitochondrial genome can encode lncRNAs [88, 89] and (ii) lncRNAs may be transcribed in the nucleus but reside in the mitochondria [90–92], a role for mitochondrial lncRNAs in the regulation of mitochondrial functions was suggested.

The first reported human mitochondrial-encoded long noncoding RNAs are SncmtRNA, a long chimeric transcript containing an inverted repeat (IR) of 820 nt covalently bound to the 5′ terminus of the mitochondrial 16S ribosomal RNA, and its two antisense transcripts (ASncmtRNA-1 and ASncmtRNA-2). All three lncRNAs are exported from the mitochondria to the nucleus and can be expressed both in normal proliferating cells and in tumor cells. Notably, both antisense transcripts are universally downregulated in cancer cells [88]. ASncmtRNA knockdown induces tumor cell apoptosis by inhibiting the expression of survivin, a member of the inhibitor of apoptosis (IAP) family, suggesting that ASncmtRNAs may take part in mitochondrial retrograde signaling [93]. In addition, although little is known regarding their potential function, several mitochondrial-encoded lncRNAs have been identified [89, 94] and could provide a new paradigm for understanding mitochondrial function.

Evidence is now emerging that several nuclear-encoded lncRNAs can execute regulatory roles in mitochondria. The lncRNA SAMMSON is predominantly localized to the cytoplasm of human melanoblasts and melanoma cells [91]. SAMMSON depletion in melanoma cells decreases the mitochondrial targeting of p32, a mitoribosome assembly and mitochondrial protein synthesis regulator, and attenuates mitochondrial protein synthesis [95], which ultimately triggers cell apoptosis. ARL2 is present in the inner membrane space of mitochondria and is an activator of ATP/ADP transporters. UCA1 can act as a competing endogenous RNA (ceRNA) and contributes to ARL2-induced mitochondrial activity by inhibiting miR-195-5p in bladder cancer [96]. The peroxisome proliferator-activated receptor γ (PPARγ) coactivator α (PGC-1α) is encoded by Ppargc1a and is a well-characterized transcriptional coactivator that plays an integral role in maintaining energy homeostasis and mitochondrial biogenesis in response to a myriad of nutrient and hormonal signals [97]. PGC-1α enhances its own transcription via an autoregulatory loop [98]. By binding directly to an R/S-rich region of the CTD of PGC-1α, the lncRNA TUG1 recruits PGC-1α protein to the Ppargc1a promoter, which enhances PGC-1α expression, leading to increased mitochondrial content, enhanced mitochondrial respiration, increased cellular ATP levels, and reduced mitochondrial ROS [99]. Bcl2 plays a key role in the mitochondrial pathway and regulates cell death by controlling the permeability of the mitochondrial membrane [100]. Tumor-suppressive MEG3 can induce the apoptosis of renal cell carcinoma cells by downregulating Bcl2 expression and thereby stimulating the mitochondrial pathway [101]; similarly, blockage of oncogenic HOTAIR induces mitochondrial calcium uptake 1 (MICU1)-dependent cell death and changes mitochondrial membrane potential by regulating mitochondrial related cell death pathway (Bcl-2, BAX, caspase-3, cleaved caspase-3, cytochrome c) [102]. LncRNA NDRG2 prevents p53 from entering the nucleus and promotes the accumulation of P53 in the mitochondria by increasing the half-life of Bad, thus promoting apoptosis in a p53-dependent manner in breast cancer cells [103]. Considering these pieces of evidence, we concluded that lncRNAs are required for mitochondrial bioenergetics and thus for the maintenance of mitochondrial energy homeostasis.

## LncRNA regulation of metabolic signaling

LncRNAs play pivotal regulatory roles in human malignancy-related metabolic reprogramming. Accordingly, the interrelation between dysregulated lncRNAs and metabolism-related factors and signaling pathways contributes greatly to the metabolic abnormalities of cancer cells, thereby promoting carcinogenesis and tumor progression (Table 1 and Figs. 2 and 3).

**Table 1:** LncRNAs deregulated in cancer through targeting the signals of metabolism

LncRNA	Location	Target	Up/down	Disease
HIF1 and MYC				
H19	Chr 11p15.5	HIF1A	Up	HCC [105]
SNHG12	/	C-MYC	Up	Breast cancer [113]
SARCC	/	AR	Down	RCC [115]
PCGEM1	Chr 2q32	C-MYC	Up	Prostatic cancer [111]
P53				
HULC	Chr 6p24.3	P53	Up	NPC [123]
HOTAIR	Chr 12	P53	Up	Breast cancer [124]
PRAL	Chr 17p13.1	P53	Down	Lung cancer [125]
PI3K/AKT/MTOR				
RP11-708H21.4	Chr 17q21	MTOR	Down	CRC [138]
UCA1	Chr 19	MTOR/STAT3	Up	CRC [63, 122]
linc00462	/	PI3/AKT	Down	HCC [139]
MALAT1	Chr 11q13	PI3/AKT	Down	Cholangiocarcinoma [177]
CASC2	Chr 10q26	PTEN	Down	CESC [7]
HULC	Chr 6p24.3	PI3/AKT	Up	Myeloid leukemia [135]
ANRIL	Chr 9q21	AKT	Up	NPC, glioma, CESC [33, 40]
AMP-activated protein kinase				
LINC00473	Chr 6q27	LKB1	Up	Lung cancer [143]
NBR2	Chr 17q21	AMPK	Down	Breast cancer [34, 42]

*Chr* chromosome, *HCC* hepatocellular carcinoma, *NPC* nasopharyngeal carcinoma, *RCC* renal cell carcinoma, *CESC* cervical cancer

### HIF1 and MYC

Hypoxia-inducible factor (HIF) complexes are transcription factors that regulate cellular gene expression in anoxic conditions. HIF1α and HIF2α are stable in anoxic environments and form heterodimers with HIF1β, which enhances the glycolytic capacity of cells by activating genes that encode transporters and most glycolytic enzymes and by reinforcing the glycolytic phenotype, through the activation of pyruvate dehydrogenase kinases (PDKs), to reduce the flow of pyruvate into the TCA cycle [1, 104]. HIF1α usually contributes to metabolic transformation in cooperation with lncRNAs. LnRNA H19 is induced by HIF1α upon oxygen deprivation in tumor cells [105]; however, H19 was efficiently repressed when HIF1-α transcriptional activity was inhibited by P53, demonstrating an important role of the p53-HIF1α-H19 pathway in hypoxia [106]. Under arsenite exposure, MALAT1, a hypoxia-inducible lncRNA, influences HIF1α protein levels via blocking HIF1α hydroxylation. In this scenario, MALAT1 disrupts HIF1α-von Hippel-Lindau (VHL) interaction and HIF1α stabilization and increases the expression of glycolytic enzymes, such as HK2 and GLUT4, thereby promoting arsenite-induced glycolysis in human hepatic cells [107]. LincRNA-p21 is a hypoxia-responsive lncRNA and can be specifically upregulated by HIF-1α under hypoxic conditions. Intriguingly, hypoxia/HIF-1a-induced lincRNA-p21 is able in turn to bind to HIF-1α and VHL and thus disrupt the VHL-HIF-1α interaction, resulting in disassociation and thereby attenuating VHL-mediated HIF-1α ubiquitination and stabilizing HIF-1α; in turn, HIF-1α increases the expression of HIF-1α responsive genes, such as those encoding the glycolytic enzymes GLUT1 and LDAH, which increases glycolysis and thus suppresses tumorigenicity [108]. The positive feedback loop between HIF-1a and lincRNA-p21 promoting glycolysis under hypoxia provides a new mechanical paradigm for the Warburg effect in human malignancies.

Myc is a canonical oncogene family that includes c-Myc, n-Myc, and l-Myc. c-Myc has been reported to promote increased aerobic glycolysis through the constitutive elevation of PFK and LDHA as well as through the expression of enzymes involved in nucleotide and amino acid metabolism [84, 85, 109]. In addition, Myc regulates glutamine metabolism and mitochondrial function by activating genes involved in mitochondrial biogenesis [17, 85]. In prostate cancer, Myc regulates glutamine metabolism by regulating the levels of SLC1A4 and SLC1A5 [110]. Prostate cancer gene expression marker 1 (PCGEM1) is an androgen-induced prostate-specific lncRNA whose overexpression is highly related to prostate cancer [111]. PCGEM1 mediates gene regulation partly through activated AR but predominantly through activated c-Myc: PCGEM1 directly binds c-Myc, promotes the chromatin recruitment of c-Myc, and enhances its transactivation activity, then increases the activity of glucose-6-phosphate dehydrogenase (G6PD), a rate-limiting enzyme of the pentose pathway, to shunt the carbon flow from glucose to ribose-5-phosphate; NADPH is generated for redox homeostasis and then participates in multiple metabolic pathways, including glucose and glutamine metabolism, the pentose phosphate pathway, nucleotide and FA biosynthesis, and the TCA cycle [111, 112]. The c-Myc-induced lncRNA SNHG12 is upregulated in triple-negative breast cancer, indicating that SNHG12 may potentially be a downstream regulator of c-Myc-regulated metabolic abnormalities [113]. Another Myc oncoprotein, N-Myc, is upregulated by lncUSMycN, leading to neuroblastoma cell proliferation via binding to the RNA-binding protein NonO [114].

In combination with the oncogenic transcription factor HIF, Myc activates the glycolytic enzymes PDK1 and LDH. As the substrate recognition module of the ubiquitin ligase complex, the VHL tumor suppressor protein (pVHL) can participate in proteasomal degradation by targeting the alpha subunits of the heterodimeric HIF transcription factor [115]. Inactivation of pVHL is a common event in clear cell renal carcinoma (ccRCC). Under hypoxic conditions, lncRNA-SARCC can physically bind and destabilize AR protein to suppress the VHL-mutant, yet it promotes wild-type RCC cell proliferation by modulating the AR/HIF-2α/c-Myc axis [116].

### TP53

The transcription factor p53 is a tumor suppressor that is downregulated in most human malignancies [117]. P53 enhances the expression of HK, TIGAR (TP53-inducible glycolysis and apoptosis regulator), PTEN, and SCO2 [118–121]. SCO2 enhances TCA, PTEN decreases the PI3K signal, and TIGAR suppresses the glycolytic activator Fru-2,6-P2. Various p53-related lncRNAs are reported to participate in tumorigenesis. For example, the lncRNA DINO (damage-induced noncoding) directly binds and stabilizes p53 protein, resulting in enhanced p53 activity [122]. MEG3 plays a tumor-suppressive role by both p53-independent and p53-dependent pathways. MEG3-activated p53 can transcriptionally activate the expression of the growth differentiation factor 15 (GDF15), thus inhibiting cell proliferation. HULC suppresses the activity of p53 and p21 to promote cell growth in nasopharyngeal carcinoma (NPC) [123]. The knockdown of oncogenic HOTAIR increases the level of p53 expression in breast cancer and thereby markedly decreases the proliferation, migration, and invasion ability of MCF-7 cells [124]. P53 was found to be downregulated in lung cancer and to be related to the tumor-suppressive lncRNA PRAL [125]. The lncRNA MIR31HG downregulates p53, promotes cell proliferation, and decreases apoptosis in non-small cell lung cancer by activating the EGFR/PI3K/AKT pathway [126]. All these findings powerfully demonstrated that lncRNAs act as regulators of p53 or its downstream effectors. Thus, the aforementioned lncRNAs likely play their role in tumor metabolism by regulating p53, and further studies are needed to clarify these specific linkages.

### The PI3K/AKT/mTOR pathway

The AKT/mTOR signaling pathway plays a pivotal role in various physiological and pathological processes, including tumorigenesis [127]. As a crucial downstream target of P13K, AKT can stimulate aerobic glycolysis by upregulating glucose transporters and glycolytic enzymes, such as hexokinase-II [128–130]. As indicated by recent studies, UCA1 activates mTOR by upregulating HK2 and increases glycolysis through both the activation of STAT3 and the repression of miR-143, thereby revealing a novel glucose metabolism regulatory pathway, UCA1-mTOR-STAT3/miR-143/HK2, in bladder cancer cells [131, 132]. Interestingly, mTOR can also be activated by CUDR, thus regulating HK2 expression through the activation of STAT3 and the repression of miR-143 [133].

Several lncRNAs have been implicated to function in the PI3K/AKT/mTOR pathway as ceRNAs. Cancer susceptibility candidate 2 (CASC2) upregulates PTEN expression and downregulates p-AKT expression by competitively binding to miR-21, thus promoting the chemosensitivity of cervical cancer cells to cisplatin [134]. HULC upregulates c-Myc and Bcl-2 by sequestering miR-200a-3p, thus activating the PI3K/AKT signaling pathway and promoting cell proliferation [135].

In addition, although the direct regulatory targets have not been elucidated, many lncRNAs have also been found to be involved in the PI3K/AKT/mTOR pathway. CRNDE promotes glioma cell growth and invasion through phosphorylation of the P70S6K-mediated mTOR pathway [136]. As mentioned above, CRNDE can increase GLUT4 transcription and contribute to glucose intake. Therefore, it is possible that CRNDE upregulates the expression of GLUT4 to promote glucose metabolism via the AKT/mTOR signaling pathway. UCA1 regulates the cell cycle via affecting CREB expression and activity through a PI3K-AKT-dependent pathway [137]. Previous data showed that ANRIL can enhance NPC progression by contributing to the expression of GLUT1 and LDHA in NPC cells [40]. The mechanism underlying the ANRIL-dependent enhancement of NPC progression may involve the ANRIL-induced phosphorylation of Akt and thus activate the mTOR pathway, which further upregulates the expression of GLUT1 and LDHA and thus promotes NPC development [138]. The lncRNA RP11-708H21.4, located in the 17q21 gene desert region, is downregulated in colorectal cancer and could regulate CyclinD1 and p27 expression by inactivating AKT/mTOR signaling, thus inhibiting tumorigenesis [139]. Linc00462 is downregulated in hepatocellular carcinoma and contributes to the inactivation of P13K/AKT signaling, thus mediating carcinogenic activity [140]. Whether RP11-708H21.4 and linc00462 participate in metabolic transformation via modulating AKT/mTOR signaling needs further comprehensive investigation.

### The AMP-activated protein kinase pathway

AMP-activated protein kinase (AMPK) is a key sensor of cellular energy, and tumor cells downregulate AMPK in order to evade restraining influences on growth and biosynthesis [141]. As a critical metabolic checkpoint, defective AMPK signaling leads to increased cell proliferation and decreased autophagy under conditions of energy stress [142]. Many types of cancer cells show AMPK signal loss, which may lead to their glycolytic phenotype [1, 141]. The tumor suppressor LKB1 is a major upstream regulator of kinases when intracellular levels of ATP are low; as such, LKB1 phosphorylates and activates AMPK, resulting in the downregulation of ATP-consuming processes and the upregulation of ATP production in the presence of AMP [143]. AMPK is activated in response to an increased AMP/ATP ratio, which causes cells to shift to an oxidative metabolic phenotype and inhibit cell proliferation. The growth-promoting role of linc00473 in lung cancer has been shown to be related to the function loss of LKB1 [144], and the lncRNA NBR2 is downregulated in breast cancer under energy stress. NBR2 executes its regulatory role by forming a feed-forward loop to potentiate AMPK activation upon glucose starvation [34]. Thus, the linc00473, NBR2, and LKB1/AMPK axis may play a pivotal role in cancer cells by regulating metabolic rearrangement.

## miRNA in cancer metabolism

miRNAs are endogenous small non-coding RNAs, 18 to 25 nt in length, that regulate gene expression [145]. Recent studies have shown that miRNAs control different aspects of energy metabolism including glucose transport and metabolism, cholesterol and lipid homeostasis, insulin production and signaling, and amino acid biogenesis [146]. The involvement of miRNAs in carcinogenesis has been well documented for almost a decade. miRNAs mediate the fine-tuning of genes involved directly or indirectly in cancer metabolism. We list the metabolic lncRNAs and miRNAs involved in regulating cancer metabolism in Table 2 [30, 33, 34, 40, 43, 48, 60, 62, 69, 71, 77–79, 81, 136, 147–162]. Obviously, both miRNAs and lncRNAs are currently known to regulate metabolic rearrangement based on various signaling pathways, as mentioned above. However, miRNAs function only at the post-transcriptional level, whereas lncRNAs exhibit great mechanical diversification, even interacting with miRNAs in cancer metabolism (Fig. 3). Studies on the roles and the underlying mechanisms of lncRNAs in metabolic rearrangement can improve the understanding of the regulatory networks of miRNAs in cancer.

**Table 2:** LncRNAs and miRNAs involved in regulating tumor metabolism

Gene	Target	Signaling	Potential functions and indication	Ref.
lncRNA and glucose metabolism				
ANRIL	GLUT1 and LDHA	PI3/AKT/mTOR	Increase glucose uptake, prognosis	[33, 40]
NBR2	GLUT1	LKB1/AMPK	Decrease glucose uptake, increases autophagy, prognosis	[34]
CRNDE	GLUT4	PI3/AKT/mTOR	Increases glucose uptake	[30, 136]
GCASPC	miR-17-3P	HIF1/MYC	Decrease pyruvate carboxylase, prognosis	[48]
NRCP	STAT1	Not mentioned	Increase glycolysis, prognosis	[49]
PVT1	HK2	miR-497/HK2 axis	Increase glucose metabolism, prognosis	[60]
H19	let-7	HIF1/MYC	Increase insulin sensitivity, enhance glucose tolerance, prognosis	[62]
MALAT1		HIF1/MYC	Increase glycolysis	[146]
microRNA and glucose metabolism				
miR-195-5p	GLUT3	LKB1/AMPK	Decrease glucose uptake	[148]
miR-210	GPD1L	HIF1/MYC	Decrease glycolysis, prognosis	[149, 150]
miR-223	c-MYC	HIF1/MYC	Increase glucose uptake, prognosis	[151]
miR-143	HK2	PI3K/AKT/mTOR	Decrease glucose metabolism	[147]
miR-21	PTEN	PI3K/AKT/mTOR	Increase glucose metabolism, prognosis	[152]
miR-326	PKM2	LKB1/AMPK	Decrease glycolysis	[153]
miR-451	CAB39	LKB1/AMPK	Increase glucose metabolism, prognosis	[156]
miR-29	MCT1	P53, PI3K/AKT/mTOR	Caused insulin resistance, prognosis	[154, 155]
LncRNA and lipid metabolism				
LncHR1	SREBP-1c and FAS	PI3K/AKT/mTOR	Decrease lipid metabolism	[69]
LNMICC	miR-190	miR-190/LNMICC/FABP5 axis	Increase fatty acid metabolism, prognosis	[71]
SRA	Not mentioned	AKT/FOXO1 axis	Decrease adipogenesis and glucose uptake	[77]
HULC	miR-9	miR-9/PPARγ/ACSL1 axis	Increase the deregulation of lipid metabolism	[76]
miRNA and lipid metabolism				
miR-122	CyclinB1	P53	Increase cholesterol synthesis and lipogenesis	[157]
lncRNA and glutamine metabolism				
TUG1	miR-145	Sirt3/GDH axis	Increase glutamine metabolism, prognosis	[81]
UCA1	miR-16	PI3/AKT/mTOR	Increase glutamine metabolism	[160]
miRNA and glutamine metabolism				
miR-23b	c-MYC	HIF1/MYC	Increase the biosynthesis of proline from glutamine	[161]

## LncRNAs and autophagy in cancer metabolism

Autophagy is an evolutionarily conserved catabolic process involving the formation of autophagosome vacuoles that engulf cellular macromolecules and dysregulated organelles, leading to their breakdown after fusion with lysosomes [163]. Much as autophagy promotes survival during starvation, cancer cells can use autophagy-mediated recycling to maintain mitochondrial function and energy homeostasis to meet the elevated metabolic demands of growth and proliferation. LncRNAs are also pivotal regulators in cancer cell autophagy. A lncRNA named lung cancer progression-association transcript 1 (LCPAT1) was shown to bind to RCC2, which upregulates autophagy and promotes lung cancer progression [164]. The lncRNA DICER1-AS1 was significantly upregulated in osteosarcoma cells. DICER1-AS1 promotes tumor proliferation, invasion, and autophagy via the miR-30b/ATG5 axis in osteosarcoma cells [165].

Cancer cells tend to constitutively activate autophagy via metabolic reprogramming [166, 167], and autophagy is also a pivotal biological process implicated in metabolic reprogramming (Fig. 2), suggesting that metabolic reprogramming and autophagy are often intertwined. Some lncRNAs have been implicated in tumor autophagy because they regulate common molecular regulatory mechanisms for both metabolic reprogramming and autophagy, such as AKT and AMPK/mTOR signaling [142, 168]. For example, linc00470 binds to FUS and AKT to form a ternary complex, anchoring FUS in the cytoplasm to increase AKT activity, which was found to inhibit the ubiquitination of HK1, affect glycolysis, and inhibit autophagy in glioblastoma cells [169]. The aforementioned metabolic lncRNAs NBR2 and ANRIL can also influence cellular autophagy by interacting with the AMPK/mTOR pathway [170, 171]. Thus, we speculated that in the process of the regulation of tumorigenesis and development by lncRNAs, metabolism and autophagy influence and promote each other to form a complex network in tumor cells. However, if specific lncRNAs regulate both autophagy and metabolism, which pathway is the predominant regulatory process in specific tumors is currently not fully clarified.

## Therapeutic potential of metabolism-related lncRNAs

Previous investigations detailing the mechanism(s) of lncRNA function in metabolic rearrangement demonstrate the potential applications of lncRNAs in novel antitumor therapies. LncRNAs are strongly associated with metabolic processes in cancer because they regulate key signaling or regulatory factors; moreover, certain lncRNAs can function as driving factors for highly tissue-specific cancer phenotypes. Thus, the rationale for using lncRNAs in metabolism is clear. Studies have reported that metformin inhibits aerobic glycolysis in cancer cells by regulating UCA1, which in turn modulates the mTOR-STAT3-HK2 pathway [172]. The upregulation of lncRNA RP11-708H21.4 inhibits migration and invasion, induces apoptosis, and enhances 5-FU sensitivity in CRC cells by inactivating mTOR signaling [139]. FA synthase (FASN) is a key lipogenic enzyme that catalyzes the terminal steps in the de novo biogenesis of FAs during cancer pathogenesis [66]. The lncRNA PVT1 has been reported to be overexpressed in osteosarcoma and to promote migration and invasion through regulating the miR-195/FASN pathway [60]. Silencing PVT1 expression restores the miR-195-mediated inhibition of FNSN, resulting in decreased tumor proliferation, migration and invasion. Therefore, PVT1 may be used as a lipid therapeutic target for the treatment of osteosarcoma. All these findings support lncRNAs as promising therapeutic targets for cancer.

We should adapt the strategies for tumor screening, diagnosis, and especially therapeutic regimens to address the metabolic reprogramming characteristics of tumors. Tumor nutritional and metabolic regulation therapies could become the main battlefield of tumor treatment. Tumor cells are characterized by high metabolic fitness and can automatically switch to other pathways when one metabolic pathway meets any obstacle, in order to avoid stress damage. Therefore, tumor metabolic regulation therapeutic regimens should be designed to sever or control multiple metabolic pathways simultaneously. Conspicuously, lncRNAs can interact with multiple molecules and/or signaling pathways (e.g., HULC with C-Myc and p53); they participate in diverse physiological and pathological processes by acting as transcriptional, post-transcriptional, or epigenetic regulators (e.g., UCA1 with HK2, C-Myc, miR-143 and miR-16); and they can target multiple metabolic processes (e.g., UCA1 in glycolysis and glutaminolysis) in a tumor simultaneously, which undoubtedly will illuminate the development and selection of therapeutic targets to prevent tumorigenesis and progression.

However, the development of lncRNA-based therapies is complicated by several common challenges in RNA therapeutics, such as the lack of reliable delivery methods and optimal dosage regimes as well as undetermined side effects. Although lncRNAs act as modulators of various human malignancies, the mechanism by which lncRNAs regulate metabolism remains largely uncharacterized. Further research will hopefully enhance the understanding of the regulatory network of cancer metabolism and provide potential targets for the development of cancer therapeutic strategies. Nevertheless, although it may currently be premature to expect lncRNA-targeted therapy to correct metabolism, the rapid development of the mechanistic modeling of lncRNA function and metabolic signaling in recent years will undoubtedly stimulate research in the field of ideal therapeutics for tumor patients with metabolic disorders in the near future.

## Conclusion

LncRNAs are well known to be able to regulate gene expression through diverse mechanisms [173]. Although the mechanisms of most lncRNAs have not been fully characterized, an elegant framework for categorizing the emerging roles of lncRNAs was recently proposed as follows: the signal archetype, a molecular signal or indicator of transcriptional activity; the decoy archetype, which binds with other regulatory RNAs or proteins to attenuate regulation; the guide archetype, which directs the localization of chromatin-modifying complex (es) and other nuclear protein (s) to specific targets to exert their effects; and the scaffold archetype, an adaptor to bring two or more RNAs and/or proteins into discrete complexes [174]. In Fig. 3, we provide an overview of currently clearly defined metabolic lncRNA function mechanisms in human tumor entities. The small number of characterized human lncRNAs have been associated with a spectrum of biological processes including transcriptional interference, the induction of chromatin remodeling and histone modifications, service as structural components, protein binding to modulate protein activity or alter protein localization, and even service as miRNA sponges. However, additional functions and detailed signaling pathways of lncRNAs remain to be clarified.

Without question, the dysregulation of lncRNAs affects multiple metabolic processes and plays a critical role in tumorigenesis and progression. Despite cumulative studies investigating the altered expression profiles of lncRNAs during metabolic rearrangement in cancer, the roles and molecular characteristics of these lncRNAs remain largely unexplored. The expression pattern and role of one lncRNA may be significantly different in different metabolic processes due to the complicated structures, specific temporal and spatial expression patterns, and tissue-specific expression of lncRNAs. Therefore, to attain a comprehensive understanding of the role of lncRNAs during tumor development, in addition to elucidating the expression patterns and functions of lncRNAs in metabolic rearrangement, further studies should also focus on structural and mechanistic characterizations. Thus far, the elucidation of the molecular mechanism underlying the Warburg effect has been of great interest in efforts to simulate tumor metabolism and to select target combinations for possible therapeutic interventions. Due to the development of software procedures, PET-CT is currently used as a clinical method for detecting cancer glucose metabolism. Metabolic tumor volume (MTV) and total lesion glycolysis (TLG) on F-18 FDG PET/CT (positron emission tomography/computed tomography) may be useful quantitative parameters for prognostic evaluation [175, 176]. However, other aspects of carbohydrate, lipid, and amino acid metabolism are rarely involved in the clinical detection and diagnosis of human malignancies. Despite decades of research, the poor understanding of tumor metabolism can clearly be attributed to the limitations of current research methods. Therefore, more comprehensive analytical strategies are desired for the study of metabolic disorders and the determination of the advantages of new strategies in different cancer diagnoses. The systematic identification and annotation of metabolism-specific lncRNA signatures and their expression patterns in tumors shows great promise for the development of accurate, noninvasive diagnostic and prognostic biomarkers. The successful development of lncRNA biotechnology and metabonomics may ultimately translate our understanding of the function of lncRNAs in cancer into a strategy for the diagnosis and treatment of cancer.

In conclusion, lncRNAs have been identified as major participants in the complex metabolic gene regulatory networks and have been found to be involved in many aspects of human malignancies. LncRNAs are crucial regulators of cell metabolism, which reinforces the importance of complementing regulatory models with the functions of lncRNAs in malignancies.

## Abbreviations

ACSL: Acyl-CoA synthase long-chain
AMPK: AMP-activated protein kinase
ANRIL: A novel large antisense noncoding RNA
ARL2: ADP-ribosylation factor-like 2
CAFs: Cancer-associated fibroblasts
CASC2: Cancer susceptibility candidate 2
ccRCC: Clear cell renal carcinoma
ceRNA: Competing endogenous RNA
CRNDE: Colorectal neoplasia differentially expressed
DDP: Cisplatin
DINO: Damage-induced noncoding
FA: Fatty acid
FABP: Fatty acid-binding proteins
FAS: Fatty acid synthase
FASN: Fatty acid synthase
Fru-2,6-P2: Fructose-2,6-bisphosphate
GCASPC: Gallbladder cancer-associated suppressor of pyruvate carboxylase
GDF15: Growth differentiation factor 15
GDH: Glutamate dehydrogenase
GLS2: Glutaminase 2
GLUTs: Glucose transporters
H3K9me1: One methylation of histone H3 on the ninth lysine
HCC: Hepatocellular carcinoma
HIF: Hypoxia-inducible factor
HK2: Hexokinase 2
ICC: Cholangiocarcinoma
LD: Lipid droplet
LDH: Lactate dehydrogenase
lncRNAs: Long noncoding RNAs
MICU1: Mitochondrial calcium uptake 1
MTV: Metabolic tumor volume
NBR2: The neighbor of BRCA1 gene 2
NDRG2: N-Myc downstream regulated gene 2
NPC: Nasopharyngeal carcinoma
NRCP: lncRNA ceruloplasmin
PC: Pyruvate carboxylase
PCGEM1: Prostate cancer gene expression marker 1
PDKs: Pyruvate dehydrogenase kinases
PFKFB2: 6-Phosphofructo-2-kinase/fructose-2,6-biphosphatase 2
PGC-1α: Peroxisome proliferator-activated receptor γ coactivator α
PKM2: Pyruvate kinase M2
PPAR: Peroxisome proliferator-activated receptor
ROS: Reactive oxygen species
RXRA: Retinoid X receptor alpha
SGLTs: Na^+^-glucose linked transporters
Sirt3: Sirtuin 3
SRA: Steroid receptor RNA
SREBs: Sterol regulatory element binding proteins
TCA: Tricarboxylic acid cycle
TERT: Telomerase reverse transcriptase
TG: Triglyceride
TIGAR: TP53-inducible glycolysis and apoptosis regulator
TLG: Total lesion glycolysis
VHL: Von Hippel-Lindau
